# Blue Light-Induced Retinal Neuronal Injury and Amelioration by Commercially Available Blue Light-Blocking Lenses

**DOI:** 10.3390/life12020243

**Published:** 2022-02-07

**Authors:** Nagarajan Theruveethi, Bang Viet Bui, Manjunath B. Joshi, Manna Valiathan, Shonraj Ballae Ganeshrao, Sivakumar Gopalakrishnan, Shama Prasada Kabekkodu, Shailaja S. Bhat, Sudarshan Surendran

**Affiliations:** 1 Department of Optometry, Manipal College of Health Professions, Manipal Academy of Higher Education, Manipal 576104, India; nagarajan.t@manipal.edu (N.T.); shonraj.bg@manipal.edu (S.B.G.); 2 Department of Optometry & Vision Sciences, School of Health Sciences, University of Melbourne, Parkville, VIC 3010, Australia; bvb@unimelb.edu.au; 3 Department of Ageing Research, Manipal School of Life Sciences, Manipal Academy of Higher Education, Manipal 576104, India; manjunath.joshi@manipal.edu (M.B.J.); shama.prasada@manipal.edu (S.P.K.); 4 Kasturba Medical College, Manipal Academy of Higher Education, Manipal 576104, India; manna.valiathan@manipal.edu (M.V.); sivakumar.g@manipal.edu (S.G.); shailaja.s@manipal.edu (S.S.B.); 5 Department of Anatomy, Manipal Campus, Melaka Manipal Medical College, Manipal Academy of Higher Education, Manipal 576104, India

**Keywords:** visual cortex neuron, caspase-3, blue light-blocking lenses, retina, light damage

## Abstract

Blue light exposure-induced retinal damage has been extensively studied. Although many in vitro studies have shown the benefits of blue light-blocking lenses (BBL) there have been few comprehensive in vivo studies to assess the effects of BBL. We investigated the influence of blue light exposure using light-emitting diodes on retinal histology and visual cortex neurons in rodents. We also considered whether retinal and cortical changes induced by blue light could be ameliorated with blue light-blocking lenses. A total of *n* = 24 (*n* = 6 in each group; control, light exposure without lenses, two different BBLs)) male Wistar rats were subjected to blue light exposure (LEDs, 450–500 lux) without or with BBLs (400–490 nm) for 28 days on a 12:12 h light–dark cycle. Histological analysis of retinae revealed apoptosis and necrosis of the retinal pigment epithelium (RPE), photoreceptors, and inner retina in the light exposure (LE) group, along with increase caspase-3 immunostaining in the ganglion cell layer (*p* < 0.001). BBL groups showed less caspase-3 immunostaining compared with the LE group (*p* < 0.001). V1-L5PNs (primary visual cortex layer 5 pyramidal neurons) demonstrated reduced branching and intersections points for apical (*p* < 0.001) and basal (*p* < 0.05) dendrites following blue light exposure. Blue light-blocking lenses significantly improved the number of basal branching points compared with the LE group. Our study shows that prolonged exposure to high levels of blue light pose a significant hazard to the visual system resulting in damage to the retina with the associated remodeling of visual cortex neurons. BBL may offer moderate protection against exposure to high levels of blue light.

## 1. Introduction

Increasingly larger segments of the population are exposed to extended periods of exposure to light from light emitting diodes (LED) [1,2]. It has been estimated that on average a person spends about 4–6 h using digital display devices, such as smartphones, laptops, and televisions that emit blue light [3,4]. Additionally, the growing uptake of efficient LEDs in the domestic setting has raised concerns, as LEDs emit more energy at shorter wavelengths compared with conventional lighting [3]. The emission spectrum of LEDs has a strong peak between 400 nm to 490 nm [5,6], with a significant proportion of the energy across this range being transmitted to the retina [7,8,9,10,11]. It is well recognized that across the visible spectrum the shorter wavelengths carry higher energy and can cause the most photothermal damage to retina cells [12,13,14].

Exposure to LEDs has been demonstrated to alter retinal structure and function, as well as other neuroendocrine systems, as evidenced by changes in cortisol levels, circadian rhythms, pupillary responses, mood, and alters behavior [15,16,17,18]. Likewise, exposure to higher levels of blue light significantly disrupts the regulation of circadian rhythms and alters behavior, as has been documented in a range of mammalian species [19,20,21,22,23,24].

Cell culture studies have demonstrated that the blue light portion of the electromagnetic spectrum (400–500 nm) induces photochemical and photothermal damage to the retina [25,26]. Specifically, exposure of cultured retinal pigment epithelial (RPE) cells to blue light compared to that emitted from digital devices causes increased free radical production and oxidative stress, which in turn leads to reduced cellular function [27,28]. Human studies and in vivo animal experimental models have demonstrated evidence of oxidative stress and damage to retinal layers when exposed to ultra-violet (UV) light from LEDs [29].

Various blue light-blocking lenses are commercially available, which seek to selectively filter out and protect the eyes from blue light. BBLs have also been proposed to improve sleep quality following the use of digital devices at night and to decrease eye tiredness and symptoms of eye strain [27,28,30,31,32,33]. Although several in vitro studies have been designed to assess the effectiveness of BBL [34], studies in cell culture systems do not recapitulate many features of prolonged exposure to high levels. Using clear cages made of blue light-blocking material used in intraocular lenses [35], a previous study demonstrated that blue light-blocking lenses reduced oxidative stress and inflammation in the RPE and choroid, induced by 20 min of exposure to very high levels of blue light (3000 lux). Whilst this in vivo study provided some insight, the light exposure employed represents acute and severe phototoxic injury. As such, in the present study, we examined the efficiency of two commercially available blue light-blocking spectacle lenses on a chronic model of moderate blue light exposure (450–500 lux) using Wistar rats. Specifically, we test the hypothesis that prolonged moderate blue light exposure impacts retinal structure and neurons in the visual cortex. We also examine the hypothesis that commercially available blue light-blocking lenses will ameliorate these effects.

## 2. Methodology

### 2.1. Experimental Setup and Animal Resources

All experimental procedures in the present study were approved by the institutional animal ethics committee (IAEC) at Kasturba Medical College, Manipal Academy of Higher Education (IAEC/KMC/02/2017). Animal handling and investigational procedures were carried out as per CPCSEA (No:94/PO/Re Bi/5/99/CPCSEA) and ARRIVE guidelines.

**Figure 1:**
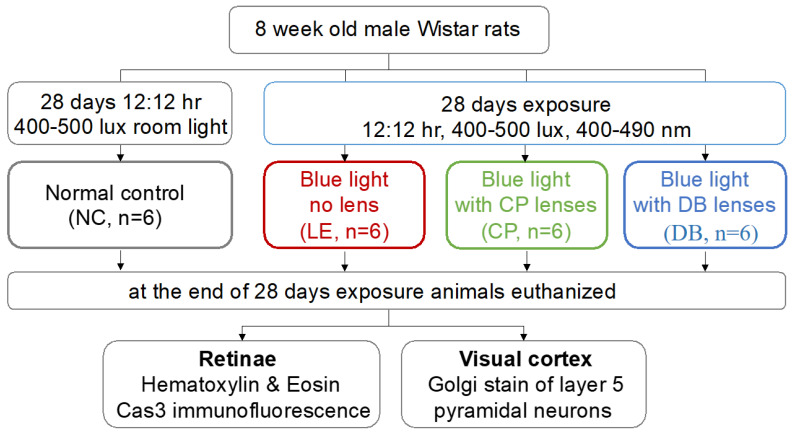
Flow chart of the methodology used in this study.

Eight-week-old male Wistar rats were procured from the central research animal facility laboratory at Manipal Academy of Higher Education (MAHE, Manipal, India). Figure 1 summarizes the experimental approach. In brief, rats were divided into four groups, with 6 animals in each group, which included a control group (NC), a blue LED light exposure group (LE), and two blue light-blocking lens protection groups, one with Crizal Prevencia (CP, Essilor, Charenton-le-Pont, France) and the other using Duravision Blue lenses (DB, Carl Zeiss, Oberkochen, Germany). The light exposure group was subjected to blue LED (400–490 nm) light (Ack LED Panels, 3W, Epistar, ES-EMBCF22L-A, InGan-series Blue LED chip, Hsinchu, Taiwan). The light source was fitted on the top of the cage (L = 100 cm, W = 70 cm and H = 50 cm) 50 cm from the cage bottom (level at the eyes was 450–500 lux). Illumination measurements (lux) were taken vertically and horizontally and averaged, to compensate for the rats’ eye anthropometrical geometry. In the blue light-blocking lens (CP and BD) groups, rats were exposed to the same light; however, BBLs (CP and DB) were fitted over the LEDs. Control group rats were maintained in a standard research laboratory lighting environment and provided with food and water *ad-libitum*. In all experimental groups, rats were exposed for 28 days, on a 12:12 h exposure cycle (at 8 a.m.).

**Figure 2:**
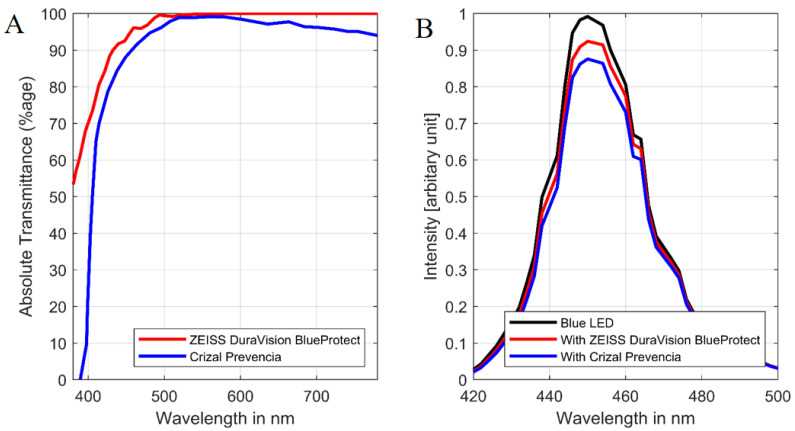
(**A**). Absolute transmittance percentage of two blue light-blocking lenses (BBLs). (**B**). Light intensity (in arbitrary unit) of the unfiltered blue LED (light emitting diodes) and with the blue light-blocking lenses in place.

Narrowband blue LED light from 400 to 490 nm was used for the experiment. Stimulus irradiance was measured at the level of the animal’s eye (50 cm from a light source above their heads) using a spectrometer (Asensetek Lighting Passport Pro, New Taipei City, Taiwan). The percentage of absolute transmittance of the blue light-blocking lenses (BBLs), CP (blue trace) and DB (red trace): wavelength emissions measured using the Zeiss Humphrey lens analyzer (LA360) (Figure 2A). The LED light source used in the experiments (black trace) is shown in Figure 2B. The blue and red traces are identical for peak wavelength but was flatter than unfiltered blue light. The absolute transmittance without BBLs (100% value of transmittance; black trace) was reduced with blue light-blocking lenses to 93.73% (DB) and 88.34% (CP) across the whole blue LED radiation spectrum.

### 2.2. Histology

After 28 days, all animals were sacrificed with a lethal dose of Pentobarbitonol (i.p. 100 mg/kg) (Virbac AH, Inc., (Westlake, TX, USA), Siegfried USA, LLC., (Pennsville, NJ, USA), Euthasol^®^) and xylazine (10 mg/kg) (Prodivet pharmaceuticals nv, Hagbenden, Proxylaz^®^, Raeren, Belgium). Eyes were enucleated using forceps (number 5) and Sklar’s blunt enucleation scissors. Immediately after enucleation eyes were placed in 4% paraformaldehyde for 7 days and retinal sections (40 μm in thickness) were cut in the sagittal plane containing the optic nerve on paraffin-embedded blocks and stained with hematoxylin and eosin (H&E) and caspase-3 immunofluorescent staining. Hematoxylin and eosin-stained retinal cross-sections were assessed by a masked histopathologist for the presence of cell atrophy, vacuolation, pyknosis, and morphological alterations (Table 1). Images were acquired from the posterior pole within 1 mm of the optic nerve. The brain was collected and immersed in a freshly prepared Golgi–Cox stain.

### 2.3. Immunofluorescence

Serial cryosections (Leica cm3050-s, Leica Microsystems, Wetzlar, Germany) 40 µm thick in the sagittal plane were mounted on gelatin-coated slides and kept at −80 °C before further processing. Sections were fixed with 2% paraformaldehyde in 0.01M phosphate buffer solution at 24 °C for 1 h, followed by incubation with 1% H_2_O_2_, 2% sodium azide, 0.1% saponin, 10 mM (4-(2-hydroxyethyl)-1-piperazineethanesulfonic acid (HEPES) in EBSS-saponin for 36 h at 24 °C in the dark. Sections were then rinsed and incubated in a moist chamber overnight at 24 °C with primary rabbit polyclonal to active + pro Caspase 3 antibody directed against cleaved caspase-3 (1:200 aliquoted 4 µL of antibody is added to 1.6 mL of PBS.TX) with 200 µL buffer (Abcam, Cambridge, UK). Sections were then washed and incubated with a secondary antibody (1:100, anti-rabbit IgG [(whole molecule) F(ab)2 fragment-Cy3 antibody, green fluorescence, Abcam)] for 16 h at 42 °C in the dark.

### 2.4. Retinal Immunofluorescence Imaging and Quantification

Caspase-3 (active + pro Caspase-3) stained retinae were imaged using a Dmi8- SP8 Confocal Microscope (Leica Microsystems, Wetzlar, Germany) with a zoom factor of 0.75 and a 63 × oil immersion objective. Excitation and emission wavelengths used were 555 nm and 569 nm, respectively. Laser power offset, gain, and other acquisition parameters were determined using isotopic control samples and were set to 0.5% for all images. These parameters were then fixed across all retinal samples. Retinal mosaics were created in Adobe Photoshop 7.0 (Mountain View, CA, USA). A total of 12 images, 2 from each eye, were analyzed in each group. Retinal layers were segmented by a masked observer, and the average intensity was calculated after subtracting the background using ImageJ software (National Institute of Mental Health 2010, License: Public Domain, BSD-2 (version 1.44k)) [36]. Examples of retinal layer segmentation can be found in Supplementary Figure S1. As they can be difficult to separate, the inner plexiform layer (IPL) was combined with the inner nuclear layer (INL). Similarly, given that in the damaged retina the outer plexiform layer (OPL) could be difficult to segment, this was combined with the outer nuclear layer (ONL). Additionally, in some cases, photoreceptors were detached from the RPE; as such, the photoreceptor layers were not included in the analysis. The brightness of each layer was then expressed relative to the average brightness of the entire retina (%). Data from images collected from the same eye were then averaged.

### 2.5. Golgi–Cox Stains for Visual Cortex Layer 5 Pyramidal Neurons (V1-L5PN)

The whole rat brain was immersed in Golgi–Cox stain (prepared 12 h before the experiment). The solution was changed every 5 days for 21 days. Tissue sections of 150 µm were obtained using a sledge microtome (RMT Series, Radial Instruments, Ambala, Cannt, India) and immersed in freshly prepared 5% sodium carbonate for 20 min. They then underwent 3 washes in 70% ethanol each for 10 min, followed by 3 washes in 90% ethanol every 10 min, 3 washes in 100% ethanol every 10 min, and 3 washes in sulphur-free xylene every 10 min, after which sections were mounted using distyrene plasticizer xylene (Merck DPX new, Sigma-Aldrich, Darmstadt, Germany).

### 2.6. V1-L5PN Image Capture Dendritic Quantification

Golgi-stained pyramidal neurons in V1 layer 5 were imaged using the 20X objective of a wide-field microscope (Motic Red 200 microscope, Kowloon, Hong Kong) and a digital camera (Moticam 580–5.0 mp with Motic Images-Plus 2.0, Kowloon, Hong Kong). Thirty photomicrographs, each separated by 5 μm in the z-plane, were collected. Photomicrographs with a calibrated Sholl’s grid (Motic software 2.0 ML, Kowloon Bay, Kowloon, Hong Kong) were projected on the laptop screen. Dendrites were manually traced in a masked fashion. For apical and basal dendrites, the number of branching points and intersections with 20 μm concentric circles up to 140 μm from the soma were quantified using Sholl’s analysis. The neurons were selected based on the following parameters: complete staining of an individual neuron, background staining, uniformity of neuronal staining, clarity in staining with dendritic spines, and any artifacts that were not taken into consideration during quantification.

### 2.7. Statistical Analysis

Statistical analysis was undertaken using R software (Massachusetts Institute of Technology (MIT), Cambridge, Massachusetts, (version 3.6.3)) [37]. Two-way ANOVA was used to compare the difference in visual cortex pyramidal neurons and one-way ANOVA was used for caspase-3 expression. Tukey’s HSD post hoc test was used to compare differences between groups.

## 3. Results

### 3.1. Retinal Histology

**Figure 3:**
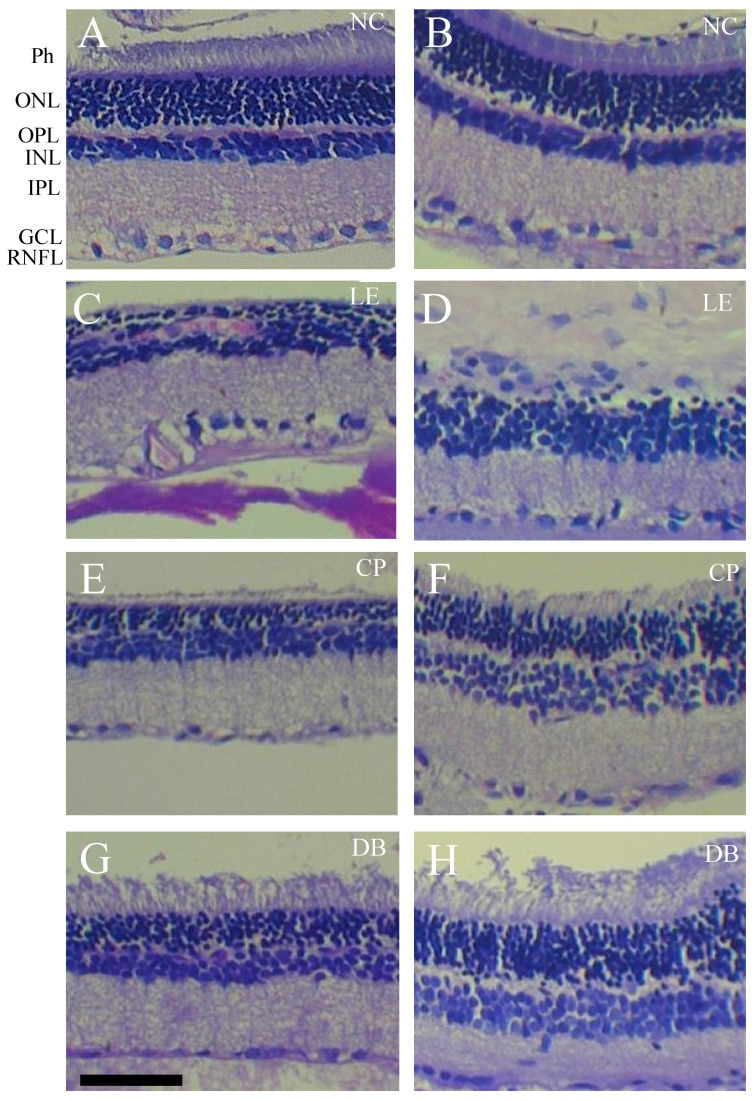
Retinal histology in normal controls (NC), blue light exposure (LE), and lens protection (CP and DB) groups after 28 days of light exposure. Representative images of hematoxylin and eosin-stained retinal cross-sections from the controls (**A**,**B**), LE (**C**,**D**), as well as CP (**E**,**F**), and DB (**G**,**H**) blue light-blocking lens groups. Scale bar = 100 μm. Photoreceptor, ganglion cell layer (GCL), inner nuclear layer (INL), inner plexiform layer (IPL), outer plexiform layer (OPL), outer nuclear layer (ONL), and retinal nerve fiber layer (RNFL).

Figure 3 shows the effect of 28 days of 12 h per day blue light exposure on retinal histology. In comparison to the normal controls (NC) group (Figure 3A,B), the light-exposed retina showed disrupted outer retinal structure, with fewer photoreceptor nuclei, loss of photoreceptor outer segments, and disrupted outer plexiform layers (Figure 3C,D). Additionally, the INL and GCL was disorganized with evidence of cell nuclei clumping and vacuoles (Figure 3D). Nucleolar damage, including disruption of the ONL, OPL, and inner retinal layers, was less evident in the lens protection groups (Figure 3E–H). Larger images are shown in Supplementary Figure S2. Pathology observations of retinal sections are summarized in Table 1.

**Figure 4:**
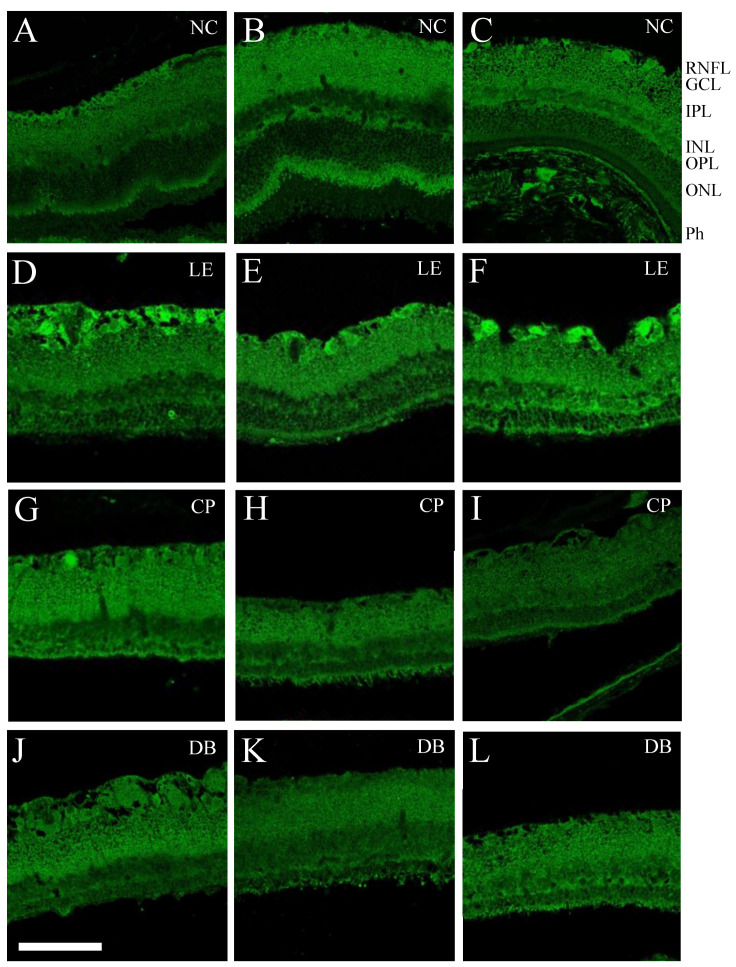
Blue light-induced caspase-3 immunostaining in retinal layers. Representative caspase -3 immunofluorescence, IgG (whole molecule) F(ab)2 fragment-Cy3 (antibody). At 28 days after light exposure, compared with controls (**A**–**C**), blue light-exposed (LE) eyes showed increased apoptosis in the GCL (**D**–**F**). This was less evident in both blue light-blocking CP (**G**–**I**) and DB (**J**–**L**) lens groups. GCL—retinal ganglion cell layer, IPL—inner plexiform layer, INL—inner nuclear layer, ONL—outer nuclear layer, OPL—outer plexiform layer, Ph—photoreceptors, and RNFL—retinal nerve fiber layer. Scale bar = 100 μm.

Figure 4 shows retinal caspase-3 immunofluorescence following 28 days of moderate blue light exposure. At this endpoint, there was increased caspase-3 immunofluorescence in the ganglion cell layer in the blue light-exposed group (LE, second row). There was less evidence of caspase-3 staining in the two BBL groups (CP, third row, and DB fourth row).

**Figure 5:**
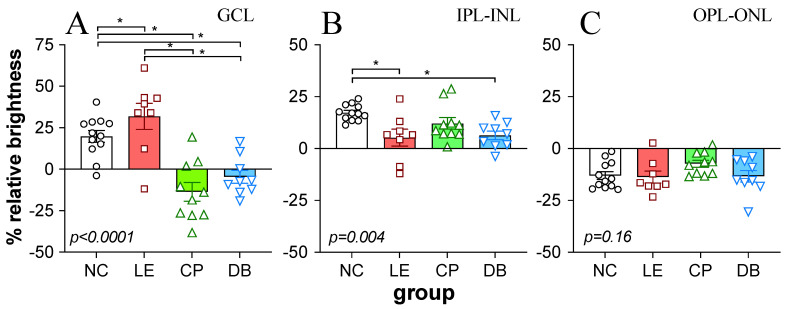
Effect of blue light exposure and blue light-blocking lenses on retinal caspase immunofluorescence. Bar plots showing signal intensity relative to total retinal intensity (%) for the ganglion cell layer ((**A**): GCL), combined inner plexiform and inner nuclear layer ((**B**): IPL-INL), and the combined outer plexiform and outer nuclear layer ((**C**): OPL-ONL). Individual values are shown. Error bars show the standard error of the mean. *p*-values in each panel indicate the significance of one-way ANOVA comparison between groups. * Indicates significant (*p* < 0.05) post hoc difference between pairs.

Caspase-3 immunostaining was quantified in the GCL as well as the IPL-INL and OPL-ONL, as summarized in Figure 5. One-way ANOVA comparisons between groups showed significant differences between groups in the ganglion cell layer (Figure 5A, *p* < 0.0001), with the blue light exposure group showing significantly higher staining than BBL groups. There was a significant difference in caspase-3 immunostaining in the IPL-INL (Figure 5B, *p* = 0.004), with LE and DB groups demonstrating significantly lower staining than the NC group. There was no difference between groups in the outer retina (OPL-ONL Figure 5C, *p* = 0.16).

### 3.2. Blue Light-Induced Changes to Visual Cortex Neurons

**Figure 6:**
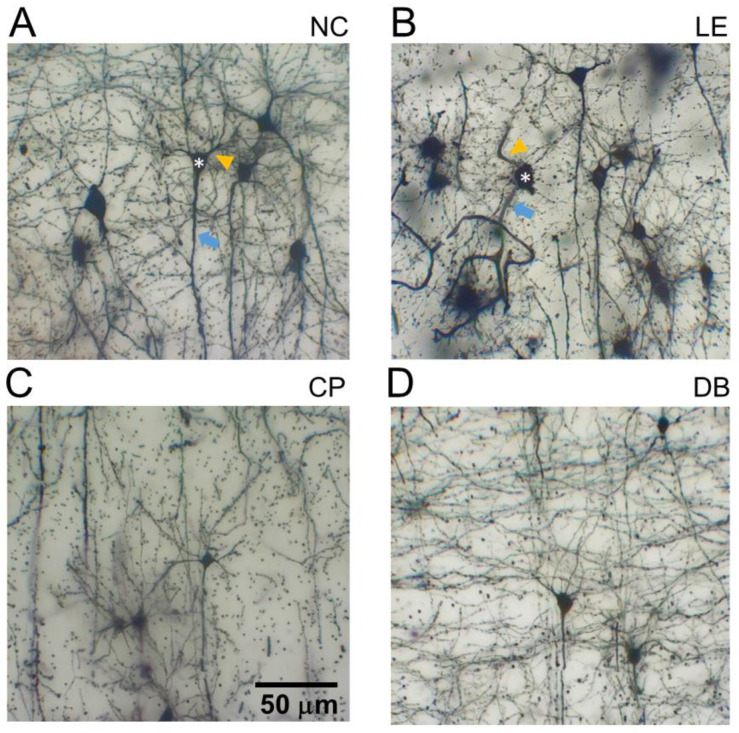
Effect of blue light exposure and blue light-blocking lenses on the morphology of pyramidal cells in the visual cortex. Representative images of Golgi-stained section of pyramidal cells in the visual cortex from an animal from control (**A**), blue light exposure ((**B**): LE) and blue light-blocking lens groups ((**C**): CP and (**D**): DB). Scale bar = 50 μm. Disruption of soma shape (*) basal (orange arrowhead) and apical dendrites (blue arrowhead) was particularly evident in the LE group.

V1-L5PN were examined 28 days following light exposure. As shown in Figure 6, blue light exposure altered dendritic morphology of both basal and apical dendrites. This disruption was particularly evident in the blue light exposure group (Figure 6B). There appeared to be less disruption in the two lens groups (Figure 6C,D).

**Figure 7:**
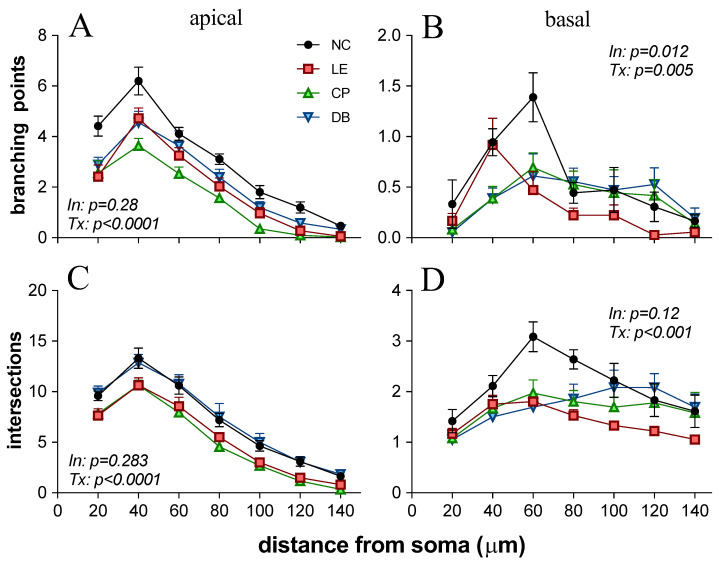
Morphology of pyramidal neurons in layer 5 of visual area 1 following blue light exposure. (**A**): Number of apical dendritic branching points as a function of distance from cell soma for all 4 groups (NC = control, LE = light exposure, CP = Crizal Prevencia, DB = Duravision Blue). Error bar indicates the standard error of the mean. (**B**): Number of basal dendritic branching points. (**C**): Number of apical dendritic intersections. (**D**): Number of basal dendritic intersections. The *p*-value for the interaction between group and distance (In) as well as for treatment difference between groups (Tx) derived from a two-way ANOVA are included.

Figure 6 confirms that blue light exposure reduced the number of branching points at all distances from the soma. Two-way ANOVA of apical branching points for all groups as a function of distance from the soma (Figure 7A), revealed no interaction (F_18,140_ = 1.18, *p* = 0.28), but there was a significant treatment effect (F_3140_ = 30.39, *p* < 0.0001). Post hoc comparison of the main effect demonstrated that all light exposure groups (LE, CP, and DB) had fewer apical branching points than the control group. For basal branching points, the LE groups showed fewer branching points further away from the soma at 60–120 mm (Figure 7B). Both CP and DB lens groups demonstrated fewer branching points closer to the soma (<60 mm) but a similar number of branching points away from the soma. Two-way ANOVA confirmed that there was a significant interaction between the treatment group and distance (F_18,140_ = 2.07, *p* = 0.012). Multiple comparisons of main treatment effects showed that there were fewer basal branching points in the LE group compared with the control group (*p* = 0.003). However, there was no difference between the CP (*p* = 0.07) and DB (*p* = 0.12) blue light-blocking lens groups compared with controls. In terms of the number of apical dendritic intersections (Figure 7C), there was a significant group difference (F_3140_ = 25.56, *p* < 0.0001), with post hoc analysis revealing that both light exposure (LE, *p* < 0.0001) and CP lens (*p* < 0.0001) groups were significantly lower than controls, whereas the DB lens group was similar to the control group (*p* = 0.98). With regards to the number of basal dendritic intersections (Figure 7D), again, there was no interaction effect, but a significant group difference (F_3140_ = 11.68, *p* < 0.0001). In comparison to the control group, light exposure (*p* < 0.0001) as well as CP (*p* = 0.001) and DB lens (*p* = 0.005) groups had significantly fewer basal dendritic intersections.

## 4. Discussion

Blue light damage to the cell can occur directly or indirectly from the release of toxic intermediaries or via inflammation [38]. Exposure to blue light causes reactive oxygen species (ROS) overproduction retinal apoptosis, and damage to mitochondria [14] which are present in high numbers, particularly in photoreceptors and in the intraocular portion of ganglion cell axons. The degree of damage depends on retinal irradiance and the duration of light exposure. Previous studies demonstrate that a high level (1000 lux or more) of acute blue light exposure for even short periods (minutes to hours) can cause damage to the RPE [9,13,14,39,40] and photoreceptors, with disruption of photoreceptor outer segments and outer nuclear layer apoptosis [14,41,42]. Consistent with these previous studies we demonstrate that chronic exposure to high levels of blue light resulted in widespread damage to the retina, including the inner retina. Specifically, in addition to gross histological disruption of the outer retina (Figure 3 and Figure 4), our results indicate that after 28 days of exposure to blue light the inner retinal ganglion cells continue to undergo apoptosis [14]. This retinal ganglion cell (RGC) apoptosis may have arisen from direct blue light damage to RGCs. Additionally, RGC death can arise as a delayed even, secondary to photoreceptor and outer retinal cell loss [43].

We found that with BBLs, there was less damage to the outer retinal layers (Figure 4 and Figure 5) consistent with a number of previous studies [30,44]. These studies show that reducing the blue light (430 nm) transmission by 50% with BBLs reduced retinal photochemical damage by approximately 80% [25,27,30,45]. We also found that in both blue light-blocking groups there was little evidence of caspase-3 fluorescence in the GCL (Figure 4 and Figure 5A). We also demonstrate for the first time that the beneficial effects of BBL on the retina also had some ameliorative effects on visual cortex pyramidal neurons.

Characterization of L5PNs in V1 revealed clear alteration of apical and basal dendritic morphology, with fewer branching points and intersection in animals chronically exposed to blue light. Damage to ganglion cell axons can lead to retrograde degeneration and death of RGC as well as the loss of anterograde of transport and Wallerian degeneration, which in turn impacts various sites of RGC termination in the brain [46]. Changes in L5PNs have been reported following retinal and ganglion cell injury, including ischemia retinal degeneration [47,48,49,50], glaucoma [51,52,53]. We show here that BBL (CP and DB) significantly reduced structural alteration in L5PNs. Specifically, BBLs increased basal branching points and DB lenses increased the number of apical intersections compared with blue light exposure. This improvement in the morphology of neurons in the visual cortex (Figure 6 and Figure 7) is consistent with a reduction in RGC apoptotic caspase-3 immunostaining (Figure 4 and Figure 5).

In our study, we considered two commercially available BBLs (CP and DB). Although there are other commercially available BBLs, we picked these two lenses because they are the most prescribed blue light-blocking lenses (data from an unpublished survey). Both CP and DB lenses provided similar benefits; however, the decline in apical intersection number was significantly improved in the DB group but not the CP group (Figure 7).

This study has some limitations. The rat lens is more transparent when compared to other mammals, and they are more susceptible to retinal damage [11]. Thus, care should be taken when extrapolating our findings to the human eye. Our study has only assessed a single time point, which was designed to model a scenario of prolonged high level blue light exposure. The spectral transmission of clear lenses generally provide over 90% transmission, down to 400 nm [54]. Given the relatively narrow band of the blue LEDs used in this study (Figure 2B) such lenses would be expected to make little difference to the blue light delivered to the control animals used in this study. A more detailed study, with more time point sampling and specific cell death assay (e.g., TUNEL), and more moderate blue light levels would help elucidate the sequence of events and help to identify the extent of retinal injury needed to drive changes in cortical neurons. Whilst we provide evidence that blue light-blocking lenses reduced retinal and V1-L5 pyramidal cell injury with high levels of blue light exposure, further studies employing electrophysiological or behavioral measures are needed to consider either of these anatomical observations in order to result in functional benefit.

## 5. Conclusions

We demonstrate that prolonged exposure to high levels of blue light causes widespread retinal injury with severe outer retinal damage, ongoing apoptosis in retinal ganglion cells, and remodeling of neurons in the visual cortex. Importantly, we demonstrate that the extent of retinal injury and cortical remodeling induced by moderate blue light exposure can be ameliorated, in part by commercially available blue light-blocking lenses.

## Figures and Tables

**Table 1:** Hematoxylin and eosin-stained retinal sections for blue LED light exposure and lens protection groups.

Layer	Damage	NC *n* = 6 (12 Sections)	LE *n* = 6 (12 Sections)	CP *n* = 6 (12 Sections)	DB *n* = 6 (12 Sections)
Ganglion cell layer	Atrophy	0	8 (66.0%)	3 (25.0%)	2 (16.0%)
	Vacuolation	0	1 (8.33%)	4 (33.0%)	4 (33%)
	Pyknosis	0	1 (8.33%)	1 (8.33%)	0
	Focal enlargement	0	6 (50.0%)	0	0
Inner plexiform layer	Atrophy	0	2 (16.0%)	0	2 (16%)
Inner nuclear nayer	Atrophy	0	2 (16.0%)	0	2 (16%)
Outer nuclear nayer	Atrophy	0	0	2 (16.0%)	0
	Decreased thickness	0	3 (25.0%)	0	0
Inner segment–outer Segment	Decreased thickness	0	8 (66.0%)	7 (58.0%)	6 (50.0%)

## Data Availability

The data presented in this study are available on request from the corresponding author.

## Supplementary Materials

The following supporting information can be downloaded at: https://www.mdpi.com/article/10.3390/life12020243/s1, Figure S1: Illustration of segmentation of retinal layer for quantification of fluorescence intensity. To account for background fluorescence, each layer is expressed relative (%) to the average brightness of the entire retina. Figure S2: Large images of retinal cross section shown in Figure 3 for a normal control (NC, A), blue light exposure (LE, B) and lens protection (CP and DB, C and D) groups after 28 days of light exposure. Scale bar = 100 μm. Photoreceptor, Ganglion cell layer (GCL), inner nuclear layer (INL), inner plexiform layer (IPL), outer plexiform layer (OPL), and outer nuclear layer (ONL), retinal nerve fiber layer (RNFL).
