# Supplementary material for: Blue Light-Induced Retinal Neuronal Injury and Amelioration by Commercially Available Blue Light-Blocking Lenses
Source: Life (Basel). 2022 Feb 7;12(2):243. doi: 10.3390/life12020243 (PMC8877890; doi:10.3390/life12020243)
Supplement: Supplementary file 1 [file life-12-00243-s001.zip › life-1525814-supplementary.pdf]

Blue Light-Induced Retinal Neuronal Injury and Amelioration by Commercially Available Blue Light-Blocking Lenses

Nagarajan Theruveethi [1], Bang Viet Bui [2], Manjunath B. Joshi [3], Manna Valiathan [4], Shonraj Ballae Ganeshrao [1], Sivakumar Gopalakrishnan [4], Shama Prasada Kabekkodu [3], Shailaja S. Bhat [4] and Sudarshan Surendran [5,*]

Supplementary Materials

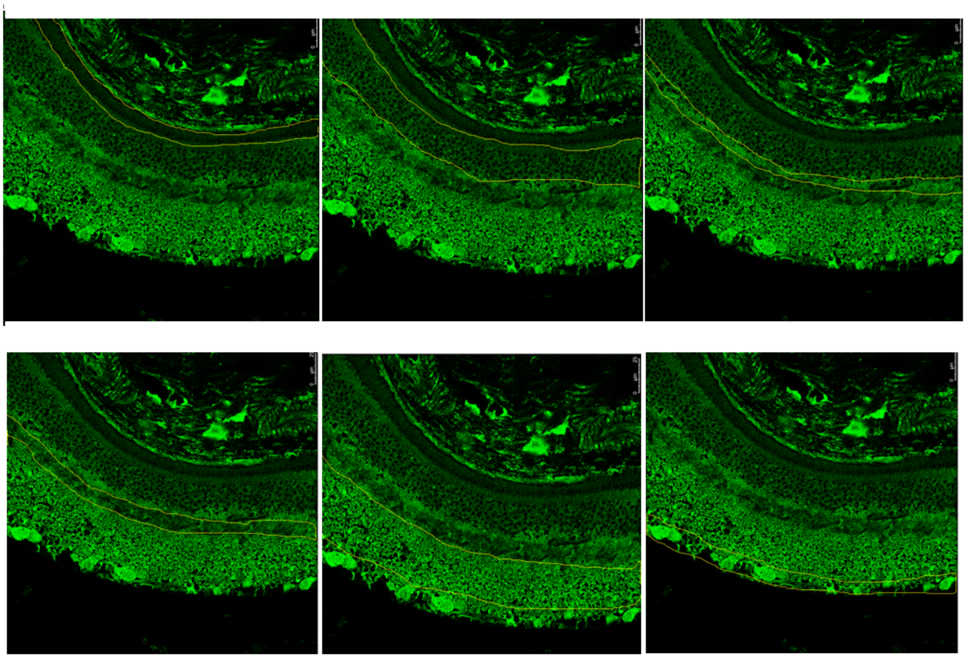

Figure S1. Illustration of segmentation of retinal layer for quantification of fluorescence intensity. To account for background fluorescence, each layer is expressed relative (%) to the average brightness of the entire retina.

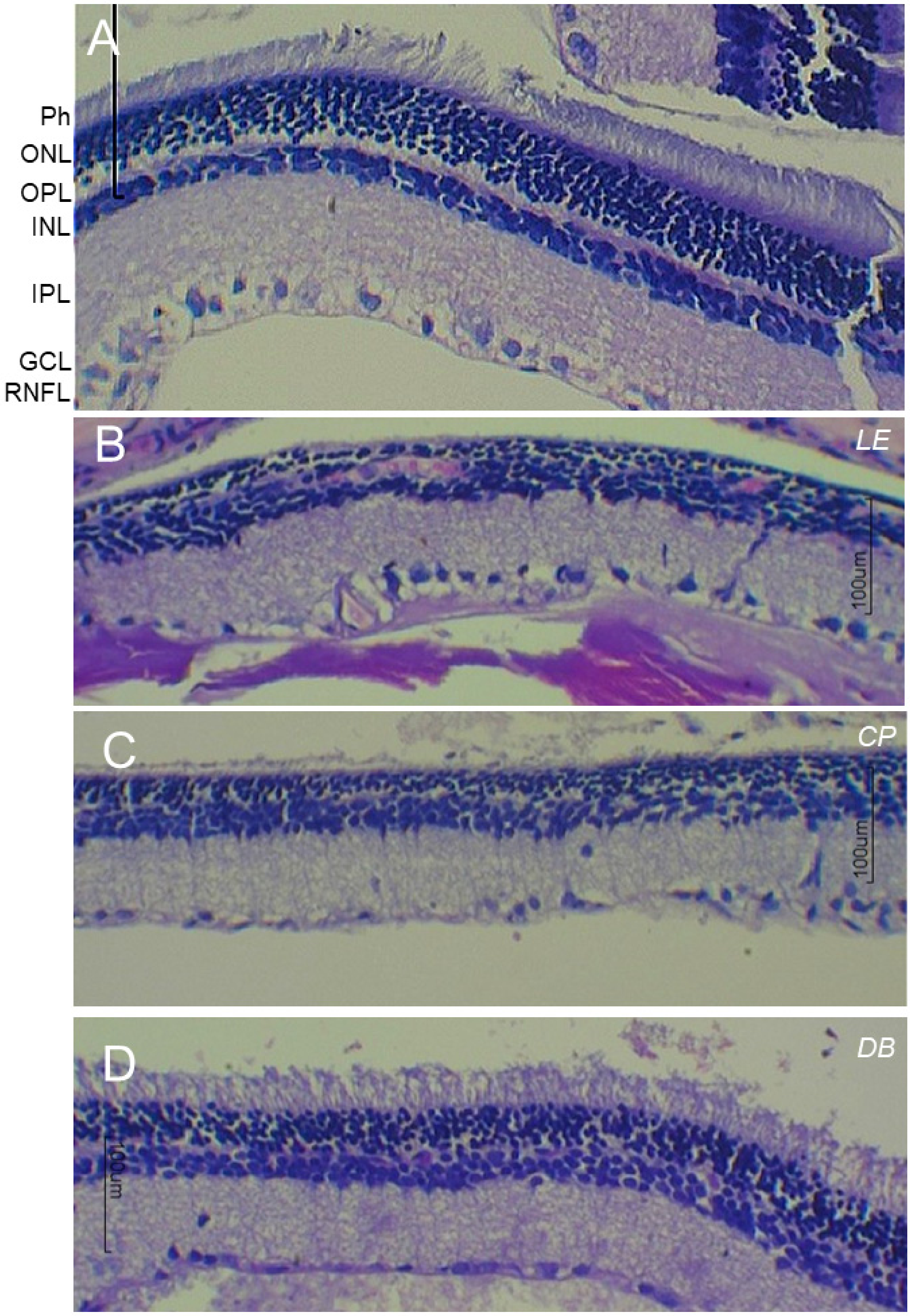

Figure S2. Large images of retinal cross section shown in Figure 3 for a normal control (NC, **A**), blue light exposure (LE, **B**) and lens protection (CP and DB, **C** and **D**) groups after 28 days of light exposure. Scale bar = 100 µm. Photoreceptor, Ganglion cell layer (GCL), inner nuclear layer (INL), inner plexiform layer (IPL), outer plexiform layer (OPL), and outer nuclear layer (ONL), retinal nerve fiber layer (RNFL).
